# Corticotroph tumour progression 37 years after bilateral adrenalectomy with temozolomide-naive response to pembrolizumab

**DOI:** 10.1530/EO-26-0064

**Published:** 2026-09-15

**Authors:** Edward Mignone, Luke Ephraums, Ian Chapman, Esther Quick, Sunita M C De Sousa, Jui Ho

**Affiliations:** ^1^ Department of Endocrinology, Flinders Medical Centre, Adelaide, Australia; ^2^ Endocrine and Metabolic Unit, Royal Adelaide Hospital, Adelaide, Australia; ^3^ SA Pathology, Royal Adelaide Hospital, Adelaide, Australia; ^4^ Adult Genetics Unit, Royal Adelaide Hospital, Adelaide, Australia

**Keywords:** corticotroph tumour progression, Cushing’s disease, bilateral adrenalectomy, aggressive pituitary tumour, immune checkpoint inhibitors, Nelson’s syndrome

## Abstract

**Summary:**

Corticotroph tumour progression (CTP) following bilateral adrenalectomy for Cushing’s disease usually emerges within the first decade; substantially delayed presentations are uncommon and longer-term surveillance recommendations remain imprecise. We report a patient presenting with CTP 37 years after adrenalectomy, representing one of the latest-onset cases in the literature. Tumour profiling demonstrated an aggressive molecular phenotype, including *ATRX* loss, *MEN1* inactivation and *CDK4* amplification, with a Ki-67 index of 10% and absent PD-L1 expression. Pembrolizumab, administered for concurrent metastatic melanoma, was associated with a rapid and sustained biochemical and radiological response, making this the fifth reported case of CTP post-adrenalectomy responding to immune checkpoint inhibitor (ICI) therapy and notably the first without prior temozolomide exposure. This case illustrates three clinically relevant points: i) lifelong post-adrenalectomy surveillance requires a structured long-term framework, ii) comprehensive molecular profiling of aggressive corticotroph tumours may inform prognosis and facilitate access to targeted therapies, and iii) ICI response in corticotrophinomas may occur despite PD-L1 negativity and without temozolomide-induced hypermutation, supporting the rationale for evaluating earlier immunotherapy in this biologically distinct subtype.

**Learning points:**

CTP is common after bilateral adrenalectomy for Cushing’s disease and requires lifelong surveillance. A pragmatic approach could be lifelong annual clinical evaluation and ACTH measurement, with MRI every 2–4 years for the first decade and then guided by annual clinical assessment and ACTH trajectory.
The role of tumour genetic testing in pituitary tumours is evolving and may be considered on an individualised basis to help predict tumour aggressiveness and potential treatment avenues in APTs.
Treatment of APTs typically involves a multimodal approach, with immune checkpoint inhibitors representing an emerging intervention when first-line therapies are ineffective.
Aggressive corticotrophinomas may require individualised treatment algorithms, with earlier consideration of immune checkpoint inhibitors.

## Background

Corticotroph tumour progression after bilateral adrenalectomy (CTP-BADx) for refractory Cushing’s disease (CD), historically termed Nelson’s syndrome, is most commonly diagnosed within the first decade following adrenalectomy, although rare late presentations have been reported (1, 2). Despite this, current guidelines do not offer a surveillance framework that extends decades post-BADx (2). By definition, CTP lesions are an aggressive corticotroph tumour and their molecular landscape is increasingly characterised, with somatic variants in *ATRX* and *TP53* now suggested as part of risk stratification frameworks (3). However, the clinical significance of less common somatic variants and the extent to which profiling should guide treatment escalation remain unknown. Immune checkpoint inhibitor (ICI) therapy represents an emerging treatment option for aggressive pituitary tumours (APTs), with corticotrophinomas appearing disproportionately represented among ICI responders relative to their prevalence within the APT population, suggesting a distinct immunobiology (4). We present an exceptionally delayed presentation of CTP 37 years after BADx, with a complex genomic profile and the first apparent temozolomide-naive response of a corticotrophinoma to pembrolizumab.

## Case presentation

A 16-year-old girl initially presented in 1987 with clinical and biochemical features of ACTH-dependent Cushing’s syndrome. Imaging revealed a normal-sized pituitary gland with an area of low attenuation, suggestive of a microadenoma, and no source of ectopic production.

After medical stabilisation, she underwent exploratory transsphenoidal surgery, which revealed no discrete lesion and no abnormal tissue on histopathology. Inferior petrosal sinus sampling confirmed CD with suspected left-sided lateralisation, prompting subsequent left hemi-hypophysectomy. Histopathology demonstrated nodular proliferation without a discrete adenoma. Persistent hypercortisolism culminated in definitive bilateral adrenalectomy. To mitigate CTP risk, she received adjuvant external beam radiotherapy (50.4 Gy in 28 fractions) to the pituitary gland. Treatment was complicated by panhypopituitarism requiring long-term hormone replacement. After two decades of surveillance, she remained clinically stable with no evidence of recurrence and was discharged from endocrine follow-up in 2007.

In 2020, she was diagnosed with stage IVa (CT4N0M1a) lung adenocarcinoma. In the context of limited access to subsidised therapy and radiologically stable disease, she elected for active surveillance. Between 2023 and 2024, surveillance imaging identified progressive sellar expansion, prompting a pituitary MRI that revealed an 18 × 17 × 20 mm sellar/suprasellar mass displacing the optic chiasm with cavernous sinus invasion (Knosp 2). At 52 years of age, 37 years after initial presentation, she had a new sellar mass, hyperpigmentation and markedly elevated ACTH at >1,750 ng/L, consistent with CTP-BADx.

**Figure 1:**
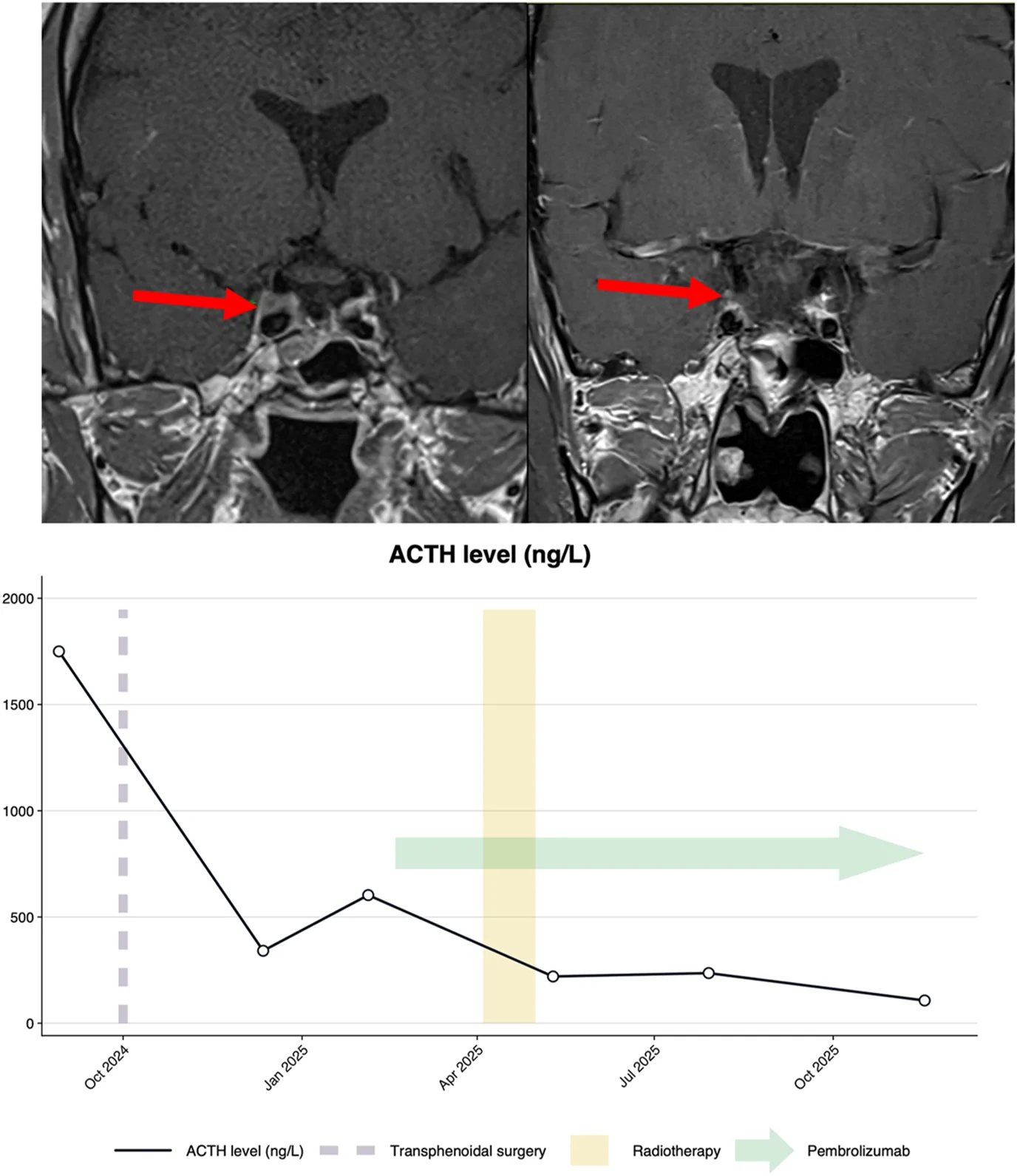
Top left: T1-weighted gadolinium-enhanced coronal magnetic resonance imaging (MRI) of the patient’s sella. Residual tumour in the right cavernous sinus (6 × 4 mm), 3 months after transsphenoidal resection, before pembrolizumab. Top right: reduction in size of tumour remnant in the right cavernous sinus (4 × 4 mm) after 15 months of pembrolizumab and 12 months after radiosurgery. Bottom: adrenocorticotropic hormone (ACTH) trend in relation to interventions. ACTH initially declined but then rose after transsphenoidal resection, followed by a significant decline at 2 weeks after radiotherapy completion and 3 months after the initiation of pembrolizumab. ACTH remains reduced at the last follow-up 15 months after the initiation of pembrolizumab therapy, which is ongoing.

**Figure 2:**
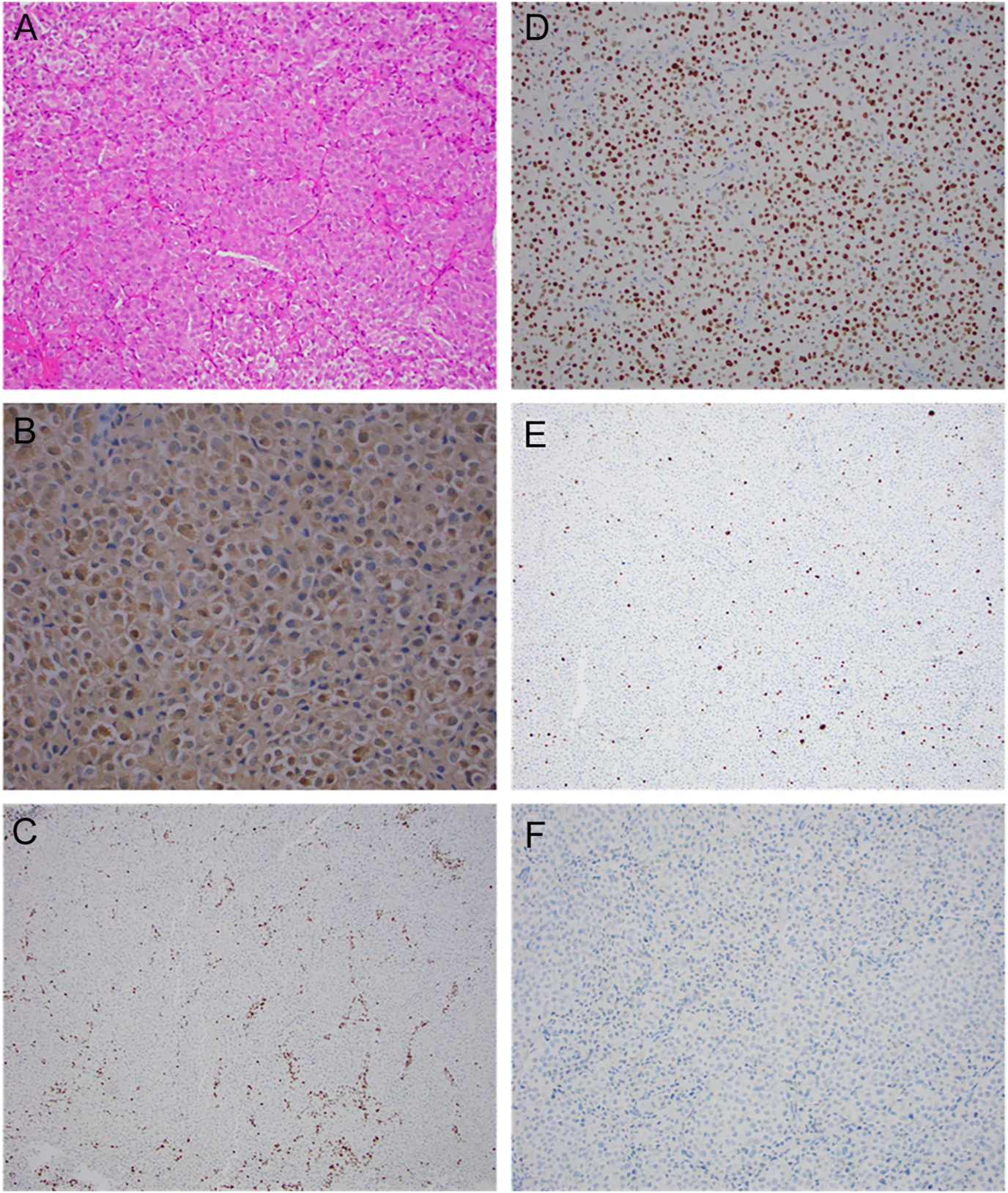
Histology images from the patient’s pituitary tumour. (A) H&E ×20 magnification depicting chromophobic cells arranged in expanded nests. Scattered mitotic figures are present. (B) Diffuse, weak ACTH expression. (C) Loss of ATRX expression by immunohistochemistry. (D) TPit immunohistochemistry labelling the tumour cells. (E) Ki-67 proliferative index 10%. (F) PD-L1 22C3 immunohistochemistry. Combined Positive Score (CPS) 0 with no labelling of tumour or inflammatory cells.

The patient underwent transsphenoidal resection with an early postoperative ACTH decline to 341 ng/L (Fig. 1). Tumour histopathology confirmed a corticotrophinoma with T-PIT and ACTH positivity, a raised Ki-67 index of 10% and a PD-L1 tumour proportion score of 0 (Fig. 2). Tumour DNA sequencing revealed somatic inactivating variants in *MEN1* (p.G225Dfs*56) and *ATRX* (p.Y587Tfs*6) and amplification of *CDK4*; germline testing was negative.

Following initial biochemical improvement, ACTH rose 6 weeks postoperatively to 603 ng/L, prompting sellar treatment with fractionated stereotactic radiosurgery (SRS) (25 Gy in 5 fractions). Two months prior to SRS, she was also diagnosed with metastatic melanoma and commenced pembrolizumab. After 3 months of pembrolizumab and just 2 weeks after radiotherapy completion, ACTH declined to 220 ng/L (Fig. 1), too soon to be plausibly attributed to radiotherapy. The biochemical response was accompanied by clinical improvement and reduced hyperpigmentation. Postoperative residual tissue in the right cavernous sinus reduced from 6 × 4 mm to 4 × 4 mm 12 months post-radiosurgery (Fig. 1). Pembrolizumab was ongoing at the last follow-up, with sustained ACTH lowering. There was no change in lung cancer volume while on pembrolizumab.

## Discussion

This presentation of CTP 37 years after BADx for CD represents one of the latest-onset cases reported in the literature. We discuss three aspects of this case: the implications for the structure of post-adrenalectomy surveillance, tumour profiling and its relevance to genomic risk stratification in APTs and the observed temozolomide-naive biochemical and radiological response to pembrolizumab.

### Implications for lifelong surveillance after bilateral adrenalectomy

CTP is diagnosed by progressive pituitary tumour growth after BADx, with rising ACTH and hyperpigmentation providing supportive evidence (2, 5). In the largest multicentre outcomes study of CTP-BADx, the median time from adrenalectomy to diagnosis was 3 years (range: 3 months to 32 years) (6), although a case occurring 39 years after BADx has been reported (1). Prophylactic pituitary radiotherapy has been suggested to reduce rates of CTP-BADx in some cohorts (7), although this remains controversial given the lack of convincing evidence of benefit and the theoretical risk that radiation-induced genomic instability may itself contribute to tumour progression (2). Current consensus recommends MRI at 3 months after adrenalectomy and annually for the first 3 years, followed by annual clinical review with ACTH measurement and MRI at decreasing frequency (2). While the principle of lifelong surveillance is endorsed, the practical structure beyond the first decade is not well defined. The present case illustrates the risk of surveillance discontinuation after prolonged apparent remission. A pragmatic approach would be lifelong annual clinical evaluation and ACTH measurement, with MRI every 2–4 years for the first decade and then guided by annual clinical assessment and ACTH trajectory thereafter.

### Molecular risk stratification in aggressive corticotroph tumours

The 2025 revised European Society of Endocrinology guidelines suggested the incorporation of molecular profiling into APT assessment, specifically suggesting somatic testing for *TP53* and *ATRX* in corticotroph macroadenomas (3). Inactivating *ATRX* variants are present in approximately 32% of corticotroph APTs/pituitary carcinomas (PCs), notably absent in non-aggressive tumours and often identifiable prior to radiotherapy and metastatic spread (3, 8). *TP53* variants often co-occur with *ATRX*-inactivating variants and independently correlate with a higher Knosp grade, parasellar invasion and disease-specific mortality (9). Somatic *MEN1* inactivation in sporadic corticotroph APTs has only been reported in isolated cases (10, 11), making its independent prognostic contribution in this context uncertain. *CDK4* amplification is a recognised driver of unrestricted cell cycle progression but has not previously been reported in pituitary adenomas. CDK4/6 inhibitors are used across several solid tumour types (12) with a single case report of potential tumour regression in a non-functioning pituitary adenoma (13). Whether this represents a therapeutically actionable target in APTs/PCs remains to be seen. Molecular profiling may uncover actionable variants and facilitate enrolment in clinical trials for APTs/PCs.

### The emerging role of immunotherapy

ICIs are currently positioned as an emerging option for APTs/PCs after temozolomide failure (3). Corticotrophinomas may be particularly ICI-sensitive relative to other pituitary adenomas. Transcriptomic profiling has shown corticotrophinomas to harbour immunologically active microenvironments, providing a mechanistic basis for checkpoint inhibitor susceptibility (14, 15). In a murine CD model, anti-PD-L1 therapy lowered ACTH levels, suppressed tumour growth and prolonged survival, with tumour-infiltrating T cells demonstrating a profile comparable to immunogenic rather than ICI therapy-resistant tumours (16). In humans, ICI-associated hypophysitis disproportionately targets corticotroph cells, with ACTH deficiency being the most common and permanent hormonal deficit (17). CTLA-4 inhibitor-associated hypophysitis is mediated in part through pituitary CTLA-4 expression and complement activation, whereas PD-1/PD-L1 inhibitors appear to exert corticotroph-selective effects through incompletely understood mechanisms (18).

Pembrolizumab in this patient was initiated for metastatic melanoma, thereby circumventing the conventional APT treatment sequence. To our knowledge, this is the fifth reported case of post-adrenalectomy CTP responding to ICI therapy (19, 20, 21, 22) and the first without prior temozolomide exposure. Previously reported corticotrophinoma ICI responders had received temozolomide prior to ICI therapy; in at least one case, temozolomide-induced hypermutation was directly implicated as a basis for enhanced immunogenicity (20). The absence of prior temozolomide exposure in our patient therefore makes this a distinct and novel observation, demonstrating that ICI responsiveness in corticotrophinomas can occur independently of temozolomide-induced hypermutation. ACTH decline occurring only 2 weeks after SRS is significantly earlier than expected for an SRS effect (2) and is therefore most consistent with an ICI-associated effect, although radiation–ICI synergy remains possible, as radiotherapy can promote immunogenic cell death and antigen presentation (23). Importantly, PD-L1 expression was absent in this tumour, as in other ICI-responsive corticotrophinomas (4, 15, 24); therefore, PD-L1 negativity should not preclude ICI consideration in aggressive corticotrophinomas.

The combination of clinical, transcriptomic and preclinical evidence suggests that corticotrophinomas may occupy a distinct immunological niche within the APT spectrum, suggesting that corticotrophinomas might warrant a treatment paradigm separate from other APTs, with an earlier threshold for ICI use rather than deferral until after temozolomide failure.

## Conclusion

CTP can present decades after apparently successful BLA, supporting structured lifelong surveillance even after prolonged remission. Comprehensive molecular profiling in this case identified a complex genomic landscape, including co-occurring ATRX and MEN1 inactivation alongside CDK4 amplification – findings that, while not directly guiding therapeutic decisions here, contribute to the emerging characterisation of aggressive corticotrophinomas and highlight the potential future relevance of routine molecular profiling in this setting. The apparent temozolomide-naive, PD-L1-negative response to pembrolizumab adds to the growing body of case-level observations suggesting that corticotrophinomas may be unusually susceptible to immune checkpoint blockade and supports the rationale for prospective studies evaluating earlier ICI therapy in this subgroup.

## Declaration of interest

The authors declare that there is no conflict of interest that could be perceived as prejudicing the impartiality of the work reported.

## Funding

This work did not receive any specific grant from any funding agency in the public, commercial or not-for-profit sector.

## Author contribution statement

All authors made individual contributions to authorship. EM and LE were involved in reviewing the case, data collection and manuscript drafting. JH, IC and SD were responsible for patient care. All authors reviewed and approved the final draft.

## Patient consent

A formal written consent for publication was obtained by JH from the patient.
